# Impact of Inclusion of Industry Trial Results Registries as an Information Source for Systematic Reviews

**DOI:** 10.1371/journal.pone.0092067

**Published:** 2014-04-17

**Authors:** Regine Potthast, Volker Vervölgyi, Natalie McGauran, Michaela F. Kerekes, Beate Wieseler, Thomas Kaiser

**Affiliations:** Institute for Quality and Efficiency in Health Care, Cologne, Germany; Johns Hopkins Bloomberg School of Public Health, United States of America

## Abstract

**Background:**

Clinical trial results registries may contain relevant unpublished information. Our main aim was to investigate the potential impact of the inclusion of reports from industry results registries on systematic reviews (SRs).

**Methods:**

We identified a sample of 150 eligible SRs in PubMed via backward selection. Eligible SRs investigated randomized controlled trials of drugs and included at least 2 bibliographic databases (original search date: 11/2009). We checked whether results registries of manufacturers and/or industry associations had also been searched. If not, we searched these registries for additional trials not considered in the SRs, as well as for additional data on trials already considered. We reanalysed the primary outcome and harm outcomes reported in the SRs and determined whether results had changed. A “change” was defined as either a new relevant result or a change in the statistical significance of an existing result. We performed a search update in 8/2013 and identified a sample of 20 eligible SRs to determine whether mandatory results registration from 9/2008 onwards in the public trial and results registry ClinicalTrials.gov had led to its inclusion as a standard information source in SRs, and whether the inclusion rate of industry results registries had changed.

**Results:**

133 of the 150 SRs (89%) in the original analysis did not search industry results registries. For 23 (17%) of these SRs we found 25 additional trials and additional data on 31 trials already included in the SRs. This additional information was found for more than twice as many SRs of drugs approved from 2000 as approved beforehand. The inclusion of the additional trials and data yielded changes in existing results or the addition of new results for 6 of the 23 SRs. Of the 20 SRs retrieved in the search update, 8 considered ClinicalTrials.gov or a meta-registry linking to ClinicalTrials.gov, and 1 considered an industry results registry.

**Conclusion:**

The inclusion of industry and public results registries as an information source in SRs is still insufficient and may result in publication and outcome reporting bias. In addition to an essential search in ClinicalTrials.gov, authors of SRs should consider searching industry results registries.

## Introduction

The aim of a systematic review (SR) is to identify the complete evidence base of the healthcare intervention under investigation, thus allowing for an unbiased evaluation of the evidence and the formulation of robust recommendations. One step in achieving this aim is to conduct a search in bibliographic databases such as PubMed and EMBASE. But this step alone may be insufficient, as such databases, besides partly containing conference abstracts, generally contain only published information. (In the context of this paper, the term “published” refers to articles published in scientific journals.) However, reporting bias has been shown between different levels of publication (journal publications, conference abstracts, reports from trial registries and results registries, as well as data on file at regulatory agencies and pharmaceutical companies) [1]–[4].

The establishment of trial registries for a priori registration of clinical trials is widely acknowledged as an effective tool to reduce their selective publication [4]–[9]. The first computerized registries were introduced in the United States in the 1960s [5]. Since then, several national and international, public and commercial registries have been created. However, mere knowledge of trial registration is insufficient, as the unbiased assessment of healthcare interventions requires access to the full information on the methods and results of the trials of interest.

On the part of the public sector, in 2007 the Food and Drug Administration (FDA) Amendments Act prescribed mandatory prospective registration, as well as disclosure of specified methods and results, for trials of drugs, biological products or devices regulated by the FDA [10], [11]. For this purpose, the US government's database ClinicalTrials.gov, primarily a trial registry, was expanded and is now the world's largest combined trial and results registry. The trial registry EudraCT was launched by the European Medicines Agency (EMA) in 2004 [12]; however, it is still under construction and the posting of results-related information has only recently become possible [13]. Selected data are publicly accessible via www.clinicaltrialsregister.eu, but no results have yet been posted (status: 13 March 2014).

On the part of the pharmaceutical industry, as early as 2002 the US member companies of the Pharmaceutical Research and Manufacturers Association committed themselves to the registration of all hypothesis-testing clinical trials at initiation and also to the timely disclosure of summary results, regardless of outcome [14], [15], and launched the meta-registry clinicalstudyresults.org in 2004. In 2005 the International Federation of Pharmaceutical Manufacturers and Associations made a similar commitment and launched ifpma.org [16]. Both commitments have since been updated [17], [18]. In addition, several pharmaceutical companies have created their own trial and results registries, albeit not always voluntarily: in the case of GlaxoSmithKline, this was the requirement of a legal settlement after the company had concealed data on antidepressants [19].

However, it is unclear whether the companies unselectively register all trials and disclose all relevant methods and results. Moreover, despite committing themselves to transparency, in numerous cases companies have tried to withhold trial data [14]. Still, industry trial and results registries might represent an important information source for unpublished information on clinical trials.

Previous research has highlighted the potential impact of searching industry results registries: A meta-analysis published in 2007 of trials of the oral antidiabetic rosiglitazone relied heavily on the results registry of the manufacturer GlaxoSmithKline, as 26 of the 42 trials were unpublished. The analysis showed that the drug “was associated with a significant increase in the risk of myocardial infarction and with an increase in the risk of death from cardiovascular causes that had borderline significance”, a finding not reported in published trials [20]. The publication of these data contributed to the market withdrawal of rosiglitazone in many countries in 2010 or to restrictions in prescription [21], [22].

The main aim of our paper was to further investigate the potential impact of the inclusion of reports from industry results registries on SRs. For this purpose, we determined what percentage of a sample of SRs of drugs did not consider industry results registries and whether the inclusion of previously unconsidered reports from these registries led to a change in the results of the SRs.

In the following text the term “registries” refers to trial registries in general; the term “results registries” specifically refers to registries containing trial results. We further distinguish between industry registries (managed by drug manufacturers or industry associations) and public registries (managed by non-profit organizations).

## Methods

### Selection of systematic reviews

We determined a sample size of 150 SRs on the basis of a pilot study. We identified SRs of drugs in PubMed using a specific systematic review filter (Table S1; search date of the original analysis: 9 November 2009). The potentially relevant citations were listed chronologically according to the most recent entry date. The titles and abstracts of citations were screened by one author. The full texts of potentially relevant citations were obtained and final eligibility was determined by one author and checked by another. Eligible documents were SRs of randomized controlled trials investigating up to 3 individual agents, used alone or in combination therapy. SRs investigating drug classes were not considered. To fulfil the classification of a “systematic” search, the search strategy had to include at least 2 bibliographic databases. If an SR failed to fulfil the above criteria, the next citation listed chronologically in PubMed was assessed until the sample size of 150 SRs was reached (backward selection). The eligible SRs were then screened by one author and checked by another to determine whether results registries of the manufacturers of the drugs under assessment and/or of industry associations had been searched or not.

### Search in industry results registries and data extraction

If industry results registries had not been searched, we searched these sources for additional trials not considered in the SR, as well as for additional data on trials already considered. For this purpose, we searched both the results registries of the manufacturers of the drugs under assessment, if available, and of the 2 main industry associations (clinicalstudyresults.org and ifpma.org). The reports retrieved were checked by one author to establish whether the corresponding trials fulfilled the inclusion criteria of the SR. The same author also checked whether the date of the report was within the search period of the SR. If no date was provided, the report was included if the completion date of the trial was at least 2 years before the search date of the SR. The reports classified as relevant were also checked by a second author.

### Data analysis

The primary outcomes reported in the SRs were reanalysed, as were the harm outcomes (adverse events and serious adverse events, withdrawals due to adverse events), if available. If no primary outcome was specifically defined in the methods section of the SR, the outcome primarily presented in the results section was used. If feasible, meta-analyses were reconducted according to the methods of the corresponding SR. In the cases where meta-analyses were not feasible, the outcomes presented in the SRs were considered to be “unchanged”.

We then compared the original results of the SRs and the reanalysed results including the data contained in the reports from industry results registries (data on additional trials or additional data on trials already included in the SR). A “change” in a result for a primary or adverse event outcome was defined as either a new relevant result or a change in statistical significance in an existing result (from non-significant to significant or vice versa). The appraisal as to whether results of the SR had changed or not was performed by one author and checked by another.

In all of the above screening, selection and appraisal steps, discrepancies between authors were resolved by consensus.

In an additional post-hoc step, we determined what proportion of SRs investigated newer drugs (i.e. drugs approved from 2000 onwards). We first checked whether the drug was listed as a generic drug in the FDA Orange Book (“Approved drug products with therapeutic equivalence evaluations” [23]). If no information was found in this source, we searched the Internet (including the EMA and FDA websites) to determine the approval status and date of approval.

### Search update

In the original analysis of 2009 we did not consider ClinicalTrials.gov, as mandatory posting of results according to the FDA Amendments Act did not apply to trials completed prior to 27 September 2007, and a 12-month time lag for posting applied to trials completed afterwards [10], [24]. The Act would therefore not have covered the vast majority of trials eligible for inclusion in SRs published in 2009. We performed a search update in 2013 to determine whether implementation of the Act had led to the inclusion of ClinicalTrials.gov as a standard information source for SRs and whether the inclusion rate of industry results registries had changed. For this purpose we screened the 20 most recent eligible SRs of drugs available in PubMed before 8 August 2013, which we had selected following the inclusion criteria of the original analysis.

## Results

The 150 SRs in the original analysis considered 125 different drugs (Table 1). Half of the SRs investigated neurological and psychiatric, antineoplastic and immunomodulating, or analgesic, antiphlogistic, and antirheumatic agents. 71 were published by the Cochrane Collaboration. 23 of the 125 drugs were newer agents.

**Table 1:** General characteristics of included systematic reviews.

	N	%
**Included systematic reviews**	**150** a	**100**
**Drug classes investigated**		
neurological and psychiatric drugs	38	25
antineoplastic and immunomodulating drugs	21	14
analgesic, antiphlogistic, antirheumatic drugs	18	12
respiratory drugs	12	8
haematopoietic drugs	10	7
gastrointestinal drugs	9	6
anti-infective drugs	8	5
cardiovascular drugs	8	5
ophthalmologic drugs	5	3
hormonal drugs	3	2
nutritional drugs	3	2
osteochondral drugs	2	1
traditional Chinese medicine	2	1
dermatological drugs	1	1
others	10	7
**Cochrane reviews**	71	47

a : All systematic reviews published in 2008 (n = 57) and 2009 (n = 93).

**Figure 1:**
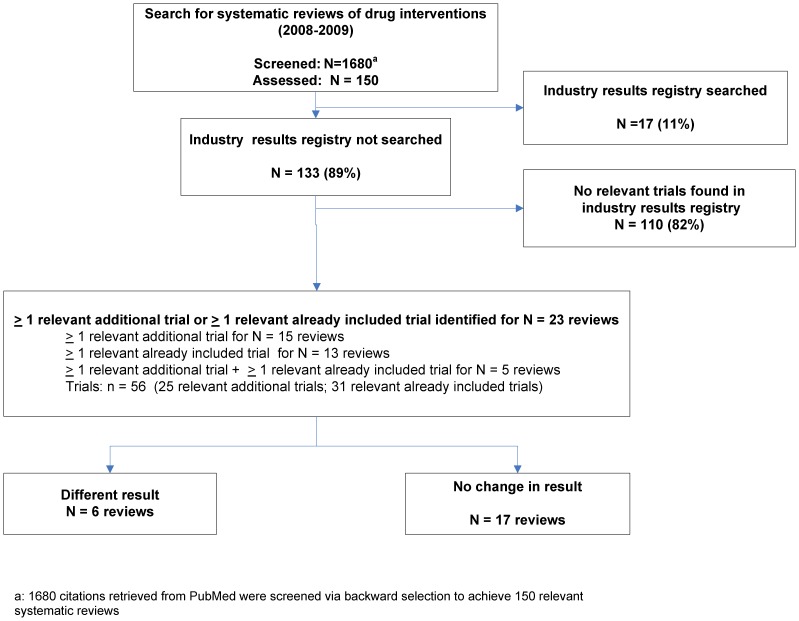
Flowchart of systematic reviews where a search in industry results registries led to a change in results.

133 of the 150 SRs (89%) in the original analysis did not search industry results registries (Figure 1); 12 of the 17 SRs that did were Cochrane reviews. For 23 of the 133 SRs we found 25 additional trials and additional data on 31 trials already included in the SRs. 18 of the 23 SRs contained meta-analyses of at least one outcome (primary outcome or one of the 3 harm outcomes). A reanalysis of the meta-analyses was feasible for at least one outcome in 10 of the 18 SRs.

The inclusion of reports from industry results registries yielded changes in existing results or the addition of new results for 6 SRs (Table 2); 17 SRs remained unchanged. All additional trials and data were identified in results registries of industry associations (in clinicalstudyresults.org for 3 SRs, in impfa.org for 1 SR, and in both for 2 SRs). No further information was identified in results registries of manufacturers. The direction of the effect on the primary outcome or harm outcome reported in the SR was statistically significantly changed in 3 SRs [25]–[27] (Table 3). Two of these changes were to the disadvantage of the test drug for an SR of valproate in schizophrenia (adverse event rate) [27] and for an SR of sibutramine in obesity (blood pressure values) [26]. One change was to the advantage of the test drug for an SR of olanzapine in bipolar disorder (withdrawal rate due to adverse events) [25].

**Table 2:** Characteristics of systematic reviews where a search in industry results registries led to a change in results.

Review	Indication	Test drug(s)	Control(s)	Trials additionally identified	Additional data identified for trials already included	Industry results registry where the additional trials/data were found^a^	Primary outcome
**Additional trials/data leading to a change in an outcome reported in the SR**							
**Schwarz 2009** [27]	Schizophrenia, schizophreniform psychoses, delusional disorder, schizoaffective psychoses	Valproate (monotherapy or in combination with antipsychotics)	Placebo (monotherapy or in combination with antipsychotics)	1 [55]	0	www.clinicalstudyresults.org	Leaving the study early
**deLemos 2008** [26]	Obesity	Sibutramine	Placebo	4 [56]–[59]	0	www.clinicalstudyresults.org www.ifpma.org	Systolic and diastolic blood pressure
**Cipriani 2009** [25]	Bipolar disorder	Olanzapine (mono or combination therapy)	Placebo alone or adjunctive to other compounds	0	4 [60]–[63]	www.clinicalstudyresults.org	Study withdrawal due to relapse of mood episode^b^
**Additional trials/data for a new comparison not reported in the SR**							
**Nakagawa 2009** [28]	Primary diagnosis of major depression	Milnacipran (monotherapy)	Other active agents in the treatment of acute major depression	1 [64]	0	www.ifpma.org	Response (reduction of at least 50% on the HAM-D or MADRS or any other depression scale, or “much or very much improved” (score 1 or 2) on CGI-improvement)
**Cipriani 2009** [29]	Primary diagnosis of major depression	Sertraline (monotherapy)	Other antidepressive agents in the treatment of acute major depression	1 [65]	4 [66]–[69]	www.clinicalstudyresults.org www.ifpma.org	Response (reduction of at least 50% on the HAM-D or MADRS or any other depression scale, or “much or very much improved” (score 1 or 2) on CGI-improvement)
**Additional data for a comparison already reported in the SR (no change in outcome)**							
**Ara 2008** [30]	Hyper-cholesterolaemia	Ezetimibe (monotherapy or combination therapy with a statin)	Statins or other lipid-regulating drugs, either alone or in combination, or no treatment	0	1 [70]	www.clinicalstudyresults.org	LDL-C^b^ ^c^

a : No further information was identified in results registries of manufacturers.

b : The designation of the primary outcome in the SR does not necessarily correspond to the operationalization of this outcome in the trials included in the systematic review (outcome in the trial included in the SR: recurrence of any affective episode; listed in Table 3).

c : If information on clinical outcomes was unavailable, the SR considered surrogate outcomes.

CGI: clinical global impression; HAM-D: Hamilton rating scale for depression; LDL-C: low-density lipoprotein cholesterol; MADRS: Montgomery–Åsberg Depression Rating Scale; SR: systematic review.

**Table 3:** Systematic reviews where a search in industry results registries led to a change in results: statistical details of results and influence on outcomes.

Review	Results of the primary outcome in the SR	Results after inclusion of reports from industry results registries^a^	Results of harm outcome(s) in the SR	Results after inclusion of reports from industry results registries	Influence of reports from industry results registries on primary outcome and harm outcome(s)^a^
**Additional trials/data leading to a change in an outcome reported in the SR**					
**Schwarz 2009** [27]	Leaving the study early	Leaving the study early	Only patients with AE were defined in the SR	Patients with AE	**Primary outcome**
					no influence
	13.5% vs. 8.0%	48.8% vs. 47.5%	84.7% vs. 76.0%	85.4% vs. 79.0%	**Harm outcome(s)**
	RR [95% CI]:	RR [95% CI]:	RR [95% CI]:	RR [95% CI]:	yes
	1.68 [0.88, 3.21]	1.00 [0.86, 1.16]	1.11 [0.98, 1.26]	1.08 [1.00, 1.16]	AEs: additional trial changed the difference between test and control groups from non-significant to significant to the disadvantage of the test drug
	p = 0.12	p = 0.97	p = 0.087	p = 0.037	
**deLemos 2008** [26]	Systolic blood pressure	Systolic blood pressure	No safety outcome was reported in the SR	No safety outcome was reported in the SR	**Primary outcome**
					yes
	WMD [95% CI]:	WMD [95% CI]:			systolic and diastolic blood pressure: additional trials changed the difference between the test and control groups from non-significant to significant to the disadvantage of the test drug
	1.57 [−0.03, 3.18]	1.49 [0.33, 2.65]			
	p = 0.05	p = 0.01			
	Diastolic blood pressure	Diastolic blood pressure			**Harm outcome(s)**
	WMD [95% CI]:	WMD [95% CI]:			no influence
	1.13 [−0.49, 2.76]	1.34 [0.36, 2.32]			
	p = 0.17	p = 0.01			
**Cipriani 2009** [25]	Recurrence of any affective episode^b^	Relapse mania or depression^b^	Withdrawals due to AE	Withdrawals due to AE	**Primary outcome**
					no influence
	olanzapine vs. divalproex:	olanzapine vs. divalproex:	olanzapine vs. placebo:	olanzapine vs. placebo:	but inconsistencies between the data in the SR and the registry report
	42.4% vs. 56.5%	43.6% vs. 60.0%	7.6% vs. 0.0%	15.6% vs. 8.8%	**Harm outcome(s)**
	RR [95% CI]:	RR [95% CI]:	RR [95% CI]:	RR [95% CI]:	yes
	0.75 [0.44, 1.28]	0.73 [0.46, 1.15]	21.22 [1.29, 349.99]	1.76 [0.95, 3.28]	withdrawals due to AEs: the SR reported a statistically significant difference between the test and control group (placebo) derived from one trial to the disadvantage of the test drug, whereas the respective registry report of the trial showed a non-significant difference
	p = 0.29	p = 0.23	p = 0.033	p = 0.076	
**Additional trials/data for a new comparison not reported in the SR**					
**Nakagawa 2009** [28]	The SR did not identify any trial for the comparison of milnacipran vs. venlafaxine	Response on HAM-D: an additional trial for the comparison of milnacipran vs. venlafaxine was found (no statistically significant difference)	The SR did not identify any trial for the comparison of milnacipran vs. venlafaxine	Patients with any AE: an additional trial for the comparison of milnacipran vs. venlafaxine was found (no statistically significant difference)	**Primary outcome**
					yes
					result for an additional comparison provided
					**Harm outcome(s)**
					yes
					result for an additional comparison provided
**Cipriani 2009** [29]	HAM-D failure to respond	HAM-D failure to respond	Treatment-emergent AEs	Treatment-emergent AEs	**Primary outcome**
					no influence,
	sertraline vs. bupropion:	sertraline vs. bupropion^c^:	sertraline vs. reboxetine:	sertraline vs. reboxetine:	but inconsistencies between data in the SR and the registry report
	OR [95% CI]:	OR [95% CI]:	no data were presented	no trial was included in the SR for this outcome; respective data were found in the additional trial (no statistically significant difference)	
	1.08 [0.80, 1.47]	0.88 [0.58, 1.33]			**Harm outcome(s)**
	p = 0.61	p = 0.54			yes
	sertraline vs. reboxetine:	sertraline vs. reboxetine^d^:			result for an additional comparison provided
	OR [95% CI]:	OR [95% CI]:			
	0.73 [0.22, 2.43]	0.83 [0.51, 1.35]	Withdrawals due to AEs	Withdrawals due to AE	
	p = 0.61	p = 0.45			
	sertraline vs. venlafaxine:	sertraline vs. venlafaxine^e^	sertraline vs. reboxetine:	sertraline vs. reboxetine:	
	OR [95% CI]:		data were presented	additional trial was identified for this comparison (2 trials were already included in the SR for this outcome) (no statistically significant difference)	
	1.07 [0.74, 1.54]				
	p = 0.72				
**Additional data for a comparison already reported in the SR (no change in outcome)**					
**Ara 2008** [30]	Results for mean change in LDL-C presented	Identical results	Patients with any AE	Patients with any AE	**Primary outcome**
			for one of the included trials no data for AE were presented	corresponding registry report of the trial contained data for the AE rates in 1 trial included in the SR: 57% vs. 56% (ezetimibe+simvastatin vs. simvastatin)	no influence
					**Harm outcome(s)**
					yes
					additional data identified on the comparison of ezetimibe+simvastatin vs. simvastatin
			Patients with any SAE	Patients with any SAE	in addition, inconsistencies between data in the SR and the registry report, but without influence
			1 vs. 2 (ezetimibe+simvastatin vs. simvastatin)	2 vs. 1 (ezetimibe+simvastatin vs. simvastatin)	
			Withdrawal due to AE	Withdrawals due to AE	
			3 vs. 2 (ezetimibe+simvastatin vs. simvastatin)	3 vs. 4 (ezetimibe+simvastatin vs. simvastatin)	

a : AEs, SAEs, treatment-emergent AEs, withdrawals due to AEs.

b . The term used for the primary outcome differs, but the operationalization of outcomes was the same.

c : Inconsistencies between the results of the original SR and the results including reports from industry results registries cannot be fully explained by the different populations analysed (intention to treat vs. randomized).

d : Inconsistencies between the results of the original SR and the results including reports from industry results registries can be explained by the inclusion of an additional trial.

e : Inconsistencies between the results of the original SR and the results including reports from industry results registries noted. However, no data shown as these inconsistencies can be explained by the different populations analysed.

AE: adverse event; CI: confidence interval: HAM-D: Hamilton rating scale for depression; LDL-C: low-density lipoprotein cholesterol; OR: odds ratio; RR: relative risk; SAE: serious adverse events; SR: systematic review; WMD: weighted mean difference.

For 2 SRs of antidepressants [28], [29] we identified additional trials or additional data for comparisons not reported in the SR. An SR of milnacipran [28] contained no data on milnacipran versus venlafaxine: we identified a trial that reported both efficacy and safety data for this comparison. For an SR on sertraline [29] we identified an additional trial presenting previously unreported treatment-emergent adverse events for the comparison of sertraline versus reboxetine; this trial also provided additional data on withdrawals due to adverse events. Finally, we identified additional data for a comparison of adverse event rates reported in an SR on ezetimibe in hypercholesterolaemia [30]. In all of the 4 comparisons above the differences between treatment groups were not statistically significant.

With regard to the provision of additional information in industry results registries, there was a notable difference between newer and older drugs: we found additional trials or data for 35% of SRs of drugs approved from 2000 onwards (8 out of 23 drugs) but for only 16% of SRs of drugs approved beforehand (15 out of 96 drugs) (Table S2).

Nine of the 20 SRs (45%) in our search update of August 2013 considered results registries, of which 8 searched ClinicalTrials.gov or a meta-registry linking to this source, and 1 searched an industry results registry. Seven of the 20 SRs were Cochrane reviews of which 6 considered results registries (Table S3).

## Discussion

### Summary of findings

Our original analysis showed that few SRs consider industry results registries and that results of some SRs change if reports from these registries are considered. The identified additional relevant trials and data on trials already included in the SRs mainly concerned drugs approved from 2000 onwards, which is not surprising, as industry results registries were not introduced until later. A search in such registries may thus be particularly worthwhile for SRs of newer drugs. Our search update showed that less than half of the SRs considered ClinicalTrials.gov as an information source, and that industry results registries were hardly considered.

### Previous research on industry and public results registries

Both our study and the meta-analysis of rosiglitazone trials already cited [20] refer to the impact of industry results registries; similar findings have been shown for public sources. As early as 1986, Simes compared results of published cancer trials and trials identified in the International Research Cancer Databank [4]. Whilst meta-analyses of published trials on ovarian cancer and multiple myeloma showed a significant survival advantage of combination chemotherapy versus treatment containing an initial alkylating agent, this advantage was absent or at least reduced in the meta-analyses of registered trials.

Two studies conducted by our Institute further highlight the relevance of information sources containing unpublished data. In 2012 we published a comparison of the completeness of reporting of information sources used for 268 trials included in 16 SRs of drugs prepared between 2006 and February 2011 [31]. Three document types were examined: journal publications, reports from industry results registries, and clinical study reports previously on file at pharmaceutical companies. Compared with journal publications, reporting quality was poorer in reports from industry results registries for methods items (P<0.001), but better for outcomes (primary outcomes and adverse events; P = 0.005); however, both sources were clearly inferior to clinical study reports. A second recently published analysis of a subsample of the above trials also showed major discrepancies between all 3 information sources regarding the completeness of reporting of several (both primary and secondary) patient-relevant outcomes such as clinical events and symptoms, quality of life, and adverse events [32]. Neither study investigated the impact of unpublished data on the actual numerical results reported in publications: however, the substantial differences in completeness of information highlight the risk of bias in published results.

### Deficits of trial and results registration

Trial registries may also provide valuable information for planned SRs or updates, as they list ongoing trials, including those soon to be completed. However, even if all SRs used registries as an information source, this would only be an approximation to the complete evidence on a topic and would thus merely reduce, but not eliminate the problem of publication bias. Firstly, as long as there is no worldwide mandatory registration of trials and trial results, the pool of trials in registries may also be incomplete, as trials may still be “hidden” on file in pharmaceutical companies and regulatory agencies. Secondly, even if a trial is registered, the information provided may be inadequate: a retrospective evaluation of 21 industry and public trial registries showed that, although compliance with the registration criteria of the World Health Organization had improved between the years 2005 and 2007, individual registry entries on study characteristics and methods were largely incomplete [33].

Despite the FDA Amendments Act of 2007, registration of results is also incomplete: a recently published analysis of 585 large randomized trials registered in ClinicalTrials.gov and completed prior to January 2009 showed that 171 (29%) remained unpublished more than 3 years later; 133 (78%) of the unpublished trials had no results available in ClinicalTrials.gov [34]. Furthermore, the Act contains a major loophole, as it was prospective and thus does not cover trials of many drugs widely prescribed in clinical practice [35]. The format originally proposed for industry results registries was based on the synopsis of a clinical study report according to ICH E3 [18], [19]. However, the ICH E3 synopsis was developed to accompany a full clinical study report and is an insufficient representation of a clinical trial. For instance, a recently published comparison of internal company synopses and journal publications on trials of the oral antidiabetic repaglinide showed that, in addition to other inconsistencies, the reporting of deaths was incomplete in both types of documents [36].

The well-known problem of selective outcome reporting in publications [37]–[40] seems to be independent of whether a trial is registered or not [41]. The registration of trial protocols (including any amendments) and of full results can help to identify this type of bias [40], [41] and is also called for in the Ottawa statement [6].

It should also be noted that industry registries may possibly contain more biased information than public ones, as biased reporting of industry-sponsored trials and meta-analyses is a well-known problem [42]–[44]. However, despite the noted potential deficits of trial registries, as long as the effort required is reasonable, SRs should at least use all available information sources, i.e. also include industry and public registries. This is all the more important as registered trials are often not published in scientific journals. This has been shown both for industry (see the rosiglitazone example above) and public registries: less than half (311 of 677, 46%) of a subsample of trials registered in ClinicalTrials.gov and completed prior to January 2006 were published 2 years after completion [45].

### Limitations

We only investigated a relatively small range of drugs and a limited number of outcomes. In addition, we did not screen the results registries of manufacturers of comparator drugs to check whether they had also conducted trials investigating the test drugs; these trials may not have been reported elsewhere. These limitations could potentially lead to an underestimation of the effect. It should also be noted that a change in the results of the SRs was defined as either the addition of new results or a change in the statistical significance of an existing result based on p-values. Although statistical significance is the usual criterion to determine whether an intervention shows an advantage or disadvantage against a comparator, statistically significant changes may not necessarily be clinically relevant and alter the conclusions of an SR.

One could argue that our study is outdated. As stated, in our original analysis we did not consider Clinicaltrials.gov, as mandatory results registration did not apply to the vast majority of trials eligible for inclusion in the SRs analysed. For instance, 49 of the 56 additional trials and data sets we identified in industry results registries had been completed before mandatory results registration became effective. As the number of industry-sponsored and publicly-funded registered trials and trial results is growing, ClinicalTrials.gov is becoming an increasingly important information source. One would thus assume that in general more recent SRs would consider this source. However, as our search update showed, only 40% of the 20 eligible SRs available in PubMed in August 2013 searched ClinicalTrials.gov or a meta-registry linking to this source, and only 5% searched an industry results registry.

With the increasing relevance of ClinicalTrials.gov, that of industry meta-registries is decreasing: clinicalstudyresults.org went offline in 2012; the expansion of ClinicalTrials.gov was cited as a reason for this measure [46]. Some reports on trials previously available on clinicalstudyresults.org are now available in manufacturer registries (see reference list), indicating that searching manufacturer registries may now be more worthwhile. The industry meta-registry impfa.org, where we identified additional trials and data for 3 SRs, is still online and thus also a potentially relevant information source.

### Further developments

In addition to the FDA Amendments Act, a further major development regarding the availability of clinical trial data is the plan by EMA to release full clinical trial data from 2014 onwards for all newly approved drugs [47], [48]. However, industry has taken legal action to prevent EMA releasing data under its current policy [49] and it is unclear whether EMA will be able to fully implement the new policy.

It is not surprising that the majority of SRs considering industry results registries in our study were Cochrane reviews. Cochrane reviews are generally of better reporting quality and typically search substantially more databases than non- Cochrane reviews [50]; in addition, the Cochrane Handbook acknowledges the increasing importance of searching trial registries [51]. However, less than 40% of Cochrane reviews have been shown to search trial registries [52] and a recent survey of over 2000 Cochrane authors reported that only about 6% and 1% obtained unpublished data from public and industry registries respectively [53]. Another guideline, the Preferred Reporting Items for Systematic reviews and Meta-Analyses (PRISMA) Statement, also recommends searching trial registries [54]. If these guidelines defined such a search as a mandatory component of any systematic search for evidence, this could provide an effective means to increase the inclusion rate of registries as an information source for SRs. For this purpose, it would be helpful if all public and industry registries were listed on a central website, to ensure complete coverage of all these sources.

### Conclusions

The inclusion of industry and public results registries as an information source in SRs is still insufficient and may result in publication and outcome reporting bias. In addition to an essential search in ClinicalTrials.gov, authors of SRs should consider searching industry results registries.

## Supporting Information

Table S1 **Search strategy for identifying systematic reviews of drugs in Medline (OVID).**
(DOC)

Table S2 **Number of drugs for which additional trial(s) or data were found in industry results registries, according to year of drug approval.**
(DOC)

Table S3 **Inclusion rate of results registries in systematic reviews of drugs identified in PubMed in August 2013 (search update).**
(DOC)
