# Supplementary material for: Impact of Inclusion of Industry Trial Results Registries as an Information Source for Systematic Reviews
Source: PLoS One. 2014 Apr 17;9(4):e92067. doi: 10.1371/journal.pone.0092067 (PMC3990559; doi:10.1371/journal.pone.0092067)
Supplement: Table S1 — Search strategy for identifying systematic reviews of drugs in Medline (OVID). (DOC) [file pone.0092067.s001.doc]

Table S1: Search strategy for identifying systematic reviews of drugs in Medline (OVID)

1. Original analysis (search date 9 November 2009)

| # | Searches |
| --- | --- |
| 1 | dt.fs. |
| 2 | "therapeutic use".fs. |
| 3 | 1 or 2 |
| 4 | exp *Therapeutic Uses/ |
| 5 | and/3-4 |
| 6 | limit 5 to (yr="2006 -Current" and "reviews (specificity)")* |

* Following Montori et al.
(Montori VM, Wilczynski NL, Morgan D, Haynes RB, Hedges T.
Optimal search strategies for retrieving systematic reviews from Medline:
analytical survey. BMJ. 2005;330(7482):68).

1. Search update (search date 08 August 2013)

| # | Searches |
| --- | --- |
| 1 | dt.fs. |
| 2 | "therapeutic use".fs. |
| 3 | 1 or 2 |
| 4 | exp *Therapeutic Uses/ |
| 5 | and/3-4 |
| 6 | limit 5 to (yr="2009 -Current" and "reviews (specificity)")* |

* Following Montori et al.
(Montori VM, Wilczynski NL, Morgan D, Haynes RB, Hedges T.
Optimal search strategies for retrieving systematic reviews from Medline:
analytical survey. BMJ. 2005;330(7482):68).
