# Supplementary material for: Impact of Inclusion of Industry Trial Results Registries as an Information Source for Systematic Reviews
Source: PLoS One. 2014 Apr 17;9(4):e92067. doi: 10.1371/journal.pone.0092067 (PMC3990559; doi:10.1371/journal.pone.0092067)
Supplement: Table S2 — Number of drugs for which additional trial(s) or data were found in industry results registries, according to year of drug approval. (DOC) [file pone.0092067.s002.doc]

Table S2: Number of drugs for which additional trial(s) or data were found in industry results registries, according to year of drug approval

|  | **Drug with first approval before 2000 n (%)** | **Drug with first approval from 2000 onwards n (%)** |
| --- | --- | --- |
| Number of drugs assessed* | 96 | 23 |
| Additional trial(s) or data found in industry results registries for 23 drugs | 15 (16%) | 8 (35%) |
| No additional trial(s) or data found in industry results registries for 96 drugs | 81 (84%) | 15 (65%) |

*In total 125 different drugs were investigated in the 150 reviews. For 6 drugs the information on the approval date was either inconsistent in the defined sources (see methods section) or the investigated drugs were herbal or traditional Chinese drugs. The presented data therefore refers to 119 with a known approval date.

n: number of drugs with approval date in the defined period
