# Supplementary material for: Impact of Inclusion of Industry Trial Results Registries as an Information Source for Systematic Reviews
Source: PLoS One. 2014 Apr 17;9(4):e92067. doi: 10.1371/journal.pone.0092067 (PMC3990559; doi:10.1371/journal.pone.0092067)
Supplement: Table S3 — Inclusion rate of results registries in systematic reviews of drugs identified in PubMed in August 2013 (search update). (DOC) [file pone.0092067.s003.doc]

Table S3: Inclusion rate of results registries in systematic reviews of drugs identified in PubMed in
August 2013 (search update)

| **Number of systematic reviews** | **N** | **%** |
| --- | --- | --- |
| **Systematic reviews identified in PubMed  in August 2013, N (%)** | **20*** | **100%** |
| **Cochrane reviews, N reviews (%)** | **7** | **35%** |
| **Systematic reviews with a search in results registries** | **9^**^** | **45%** |
| Search in public results registries   - At least in ClinicalTrials.gov - Meta-registry linking to ClinicalTrials.gov (who.int/trialsearch or controlled-trials.com) | 8  6  2 | 89% |
| Search in industry results registries   - Manufacturer results registry (Novartis: novctrd.com/ctrdWebApp/clinicaltrialrepository/public/main.jsp) | 1 | 11% |

*All systematic reviews published in 2012 (n=3) or 2013 (n=17).

- **Cochrane reviews: 5 searched ClinicalTrials.gov or a meta-registry linking to ClinicalTrials.gov and 1 searched an industry results registry.
